# Medical Schools

**Published:** 1857-12

**Authors:** S. C. Nott

**Affiliations:** Professor of Anatomy in the University of Louisiana


*Medical Schools:* By S. C. Nott, M. D., Professor of Anatomy in the University of Louisiana.

Having been recently called to the professorship of Anatomy in the University of Louisiana, my mind has, necessarily, been attracted more closely to the subject of medical education, and with the view of posting myself up fully in all the latest improvements for teaching anatomy, and of comparing the advantages and disadvantages of various points, I have occupied the past summer in visiting many of the more prominent schools in the United States.

The present number of this Journal is about going to press in a few days, and although I have been but twenty-four hours in the city and have much else to occupy me in making arrangements for a change of residence, I have promised the editors that I will throw together hastily, the impressions which have been made on my mind, hoping at some future day to return to the subject more in detail.

It has been a prevailing idea, that the climate of New Orleans and other southern cities is an insuperable barrier to the full success of medical schools, and I confess that this prejudice has been very strongly rooted in my own mind. Though familiar with the fact that the history of medicine from the earliest epoch to the sixteenth century belongs almost exclusively to hot climates, yet the medical schools of temperate latitudes in our day, have so far outstripped the ancient seats of learning, that I had began to think that the sceptre of science, as of political power, had passed into the hands of those northern people who have of late been governing the world.

When I turned my mind more closely to the subject, and analyzed the causes of the downfall of ancient schools, I soon found that there was something at work more baneful than *climate*, viz: misrule and indolence. Egypt was the most ancient seat of medical science, both before the conquest of the Greeks and during the existence of her famous Alexandrian school. If we follow the map around the Mediterranean Sea, we find that the march of medical science for ages was still exclusively through southern climes—Ar bia, Greece, Rome, and in the middle ages, Italy, in succession held all of medical science known to the world. But the beastly Turk in Egypt, Arabia and Greece, and the despotic Austrian in classic Italy, have crushed out not only medical, but every other department of science and literature. If there are no Hippocrateses, Galens or C[illegible]uses—no Homers or Virgils—it is not because genius is extinguished in these races, but it is because despotism has placed its foot on mind and body too.

The great difficulty, to my mind, against which southern schools had to contend, was the difficulty of pursuing dissections during our mild winters, for not only is the putrefaction of bodies disgusting and unwholesome, but it is inimical to the successful pursuit of dissection. For without the aid of antiseptics, a body will keep in Boston during the winter, a month, while in Charleston or New Orleans, there are times when you could not work on one for a week.

Anatomy is the ground work of the science and art of medicine, and other things being equal, the student should always seek that school where anatomy is best taught. It is vain to talk of learning physiology, pathology, surgery, obstetrics, or practice of physic without anatomy, and it is infinitely better to have no doctors at all than to have incompetent ones.

Those climates and those localities alone which afford the fullest anatomical advantages, can meet the requirements of the profession, and the student who is really conscientiously seeking to prepare himself for the arduous and responsible duties which await him, should keep his eyes steadily on this point and not be gulled by newspaper puffs, circulars, and false promises. I repeat to the medical student, if you desire to effect anything useful or noble in our profession, go where you can learn thoroughly anatomy.

My doubts are now entirely removed with regard to the appropriateness of the climate of New Orleans for anatomical investigation, for we can preserve bodies perfectly in any weather, an indefinite period of time. I have just returned from Philadelphia, where I have received from my obliging friend Professor Leidy, much valuable information, and seen bodies preserved during mid-summer by means of antiseptics, as perfectly as they could be by the climate of Moscow or St. Petersburg. I dissected myself, in the private room of Professor Leidy, a body prepared under his direction, for two weeks, during the hot weather of September, and it was as sound and free from putrid odor the last day as it was the first—nay, more, the brain, nerves, and other soft tissues, were rendered much more firm than natural and the rapidity and minuteness of dissection was much facilitated.

No city in the United States can at all compare with New Orleans in point of abundance of material for the anatomical student. We can command bodies for dissection to any extent at an expense far below any institution I am acquainted with, in fact, at the moderate cost of transportation of subjects to our rooms.

The facilities afforded for clinical instruction in the city of New Orleans are not equalled anywhere in the United States, and surpassed, if at all, by few places in the world. I am writing in haste and have not time to look up statistics of later date, but have beside me the "Report of the Board of Administrators of the Charity Hospital for 1850," in which the statement is made that the number of admissions into this institution for the years 1850–51 were respectively 18,476 and 18,420. There has been no change in the administration since that time, and the usefulness and interest of this great establishment has never flagged. The number of patients fluctuates from six or seven hundred up to fifteen hundred, and when we state that it is under the superintendence of such "ministering angels" as the *Sisters of Charity*, nothing need be added to illustrate the internal management of this charity.*

We have already stated that the history of medicine, down to the middle ages, is to be sought exclusively among the people of warm climates; in fact, it is not until the seventeenth century that Germany, France and Great Britain began to take an active part in its cultivation.

Although we must gratefully acknowledge our indebtedness to Hippo-

---

*The number of medical cases treated during the last year (1856) in the Hospital, was about 6,450; the surgical patients numbered 2,450; and the obstetrical cases, and those of special diseases of women and children about 500." *Annual Circular, Medical Department, University La.*

crates, Galen, Celsus and other great names of antiquity, so great were the prejudices and difficulties which opposed the study of anatomy, that it was not till the sixteenth century that dissections of the human body were common, and it is to classic Italy, with a climate similar to ours, that we must turn not only for the revival of medicine, but for that solid advancement in anatomy which has laid the ground work of rational medicine. The schools of Padua, of Pisa, of Rome, and of Florence will long be pointed to as a bright galaxy in our history; and the names of Fallopius, Eustachaius, Arantius, Cæsalpinus, Fabricus, Spigelius, Vasalius, of Scarpa, Morgagni, Mascagni, Valsalva, Malpighi, Spallanzani, and many other Italian names will stand out in honorable relief, as long as medical history is written and read. These laborers too have done their work in a climate not more favorable to anatomy than ours, and that without the immense advantages afforded us by modern chemistry in preserving bodies.

The museum of the University of Louisiana is unrivalled in this country. We need only state that besides numerous models from the best artists in France and England, we have duplicated the celebrated collection of the Grand Duke of Tuscany at the Academy of Anatomy in Florence, which is unsurpassed—the latter collection alone gives more than 350 dissections, representing in detail the whole anatomy of the human system. together with many illustrative models of comparative anatomy.

But there is another view of the subject which should give great weight to the value of southern schools. The fact is well known that the diseases of warm and cold climates differ as much as do their fauna and flora. It has been said that a young man should not go to a medical school with the expectation of learning the practical details of his profession, but simply to ground himself well in those fundamental principles which are to guide him in after life; and that this kind of knowledge can only come with the routine of a bedside experience. This is to some extent true, for in reality a medical student, after the ordinary routine of instruction, has learned little more than *how to learn*; but it is absurd to say, other things being equal, that there is not an advantage in a southern over a northern education, to those who are to practise in malarial districts. Those professors who have been for twenty or thirty years treating daily the diseases of the south, should certainly be more competent to instruct southern young men than teachers who have little or no experience with our diseases. I have never seen a lecture or essay from the pen of a northern lecturer on remittent yellow fever that I could read half through, for I could see internal evidence at every step, that he was talking of things at secondhand, and about which he knew nothing. A northern lecturer would laugh at the idea of a New Orleans physician attempting to teach him anything about pneumonia, so different is the type of the disease in the two latitudes.

Still I would say to the medical student, go to that school where you can learn most anatomy, physiology and pathology, for these are the ground works which without, a really enlightened and practical physician cannot be made, north or south, and it would be far better to annihilate the medical profession at once, than to fill the country with licensed quacks who are a curse instead of a blessing to society.

If, then, New Orleans possesses all the appliances for medical instruction in such extraordinary profusion, why, let me ask, can she not rear up a medical school, or schools equal to any in America? It is not my desire to make invidious comparisons, or to say unkind or ungenerous things of other schools. I should depise myself if I could be swayed by such feelings or such motives. The field of science is a Republic equally open to all; and with the population of the United States rapidly increasing, as it is, there will be room enough, and patronage enough for all those schools so located as to have the real substantial advantages necessary for medical instructions All that should be asked by a school is "day light and fair play," and I for one shall always be ready to give the right hand of fellowship to those who pursue science, not filthy lucre or false fame, but who, like men of honor, are willing to work in the cause of humanity, under the guidance of a fair, gentlemanly and scientific spirit.

New Orleans at this moment affords ample facilities for instructing 1000 medical students; facilities are increasing yearly; and so far from depreciating honest rivalry and emulation, I shall be glad to see it, for the great ends of science and humanity will by such means be the most surely attained.

Why, let me again ask, should not New Orleans become one of the leading seats of medical instruction in the United States? There is but one answer to this question—*all depends upon the medical teachers of New Orleans*—their energy, industry, and fidelity to the great trust reposed in them—if the schools fail it will be from the indolence, incompetence, and want of character of our teachers.

In this respect we may learn useful lessons from some of our brethren of the North. I have spent a considerable portion of the past summer in Philadelphia, the field of my early professional studies, and I still feel a pride in my alma mater which has been the mother of medical science in

America. I could not but admire the patient and enlightened industry with which this school, with its worthy rival (Jefferson School) have been laboring on from year to year in the good cause. May they long preserve the high position which they have so nobly earned, and remain as beacons to stimulate our exertions in the cause of science.—*N. O. Medical and Surgical Journal.*

